# Motion of a Legged Bidirectional Miniature Piezoelectric Robot Based on Traveling Wave Generation

**DOI:** 10.3390/mi11030321

**Published:** 2020-03-20

**Authors:** Jorge Hernando-García, Jose Luis García-Caraballo, Víctor Ruiz-Díez, Jose Luis Sánchez-Rojas

**Affiliations:** Microsystems, Actuators and Sensors Group, Universidad de Castilla-La Mancha, E-13071 Ciudad Real, Spain; JoseLuis.GCaraballo@uclm.es (J.L.G.-C.); Victor.Ruiz@uclm.es (V.R.-D.); JoseLuis.SAldavero@uclm.es (J.L.S.-R.)

**Keywords:** robot, piezoelectric, miniature, traveling wave, leg

## Abstract

This article reports on the locomotion performance of a miniature robot that features 3D-printed rigid legs driven by linear traveling waves (TWs). The robot structure was a millimeter-sized rectangular glass plate with two piezoelectric patches attached, which allowed for traveling wave generation at a frequency between the resonant frequencies of two contiguous flexural modes. As a first goal, the location and size of the piezoelectric patches were calculated to maximize the structural displacement while preserving a standing wave ratio close to 1 (cancellation of wave reflections from the boundaries). The design guidelines were supported by an analytical 1D model of the structure and could be related to the second derivative of the modal shapes without the need to rely on more complex numerical simulations. Additionally, legs were bonded to the glass plate to facilitate the locomotion of the structure; these were fabricated using 3D stereolithography printing, with a range of lengths from 0.5 mm to 1.5 mm. The optimal location of the legs was deduced from the profile of the traveling wave envelope. As a result of integrating both the optimal patch length and the legs, the speed of the robot reached as high as 100 mm/s, equivalent to 5 body lengths per second (BL/s), at a voltage of 65 V_pp_ and a frequency of 168 kHz. The blocking force was also measured and results showed the expected increase with the mass loading. Furthermore, the robot could carry a load that was 40 times its weight, opening the potential for an autonomous version with power and circuits on board for communication, control, sensing, or other applications.

## 1. Introduction

Control of locomotion in artificial structures is paramount for development in many disciplines. After the breakthroughs at the macroscale, there is a considerable interest in the scientific community for the development of miniature locomotion systems for multi-functional millimeter-to-centimeter scale robotic platforms capable of performing complex tasks for disaster and emergency relief activities, as well as the inspection of hazardous environments that are inaccessible to larger robotic platforms [1]. Miniaturization in the field of locomotion would result in advantages, such as smaller volume and mass, access to restricted volumes, interaction with same-sized targets, lower cost, profit of scaling laws, and so on [2].

When considering mechanisms of locomotion at the miniature scale, the use of wave-driven structures stands out because of its simplicity, reduced thickness, and low cost. The nature of the waves might be either traveling or standing. Furthermore, the generation of such waves can be easily accomplished with the help of piezoelectric materials, which can be integrated onto the structures during the fabrication process. A clear example of the potentiality of waves for locomotion are ultrasonic motors [3,4,5]. A history of commercial success was achieved using circular traveling wave-based stators transmitting its energy to a rotor [6,7], with the capability of bidirectional movement. Standing-wave-based motors were also reported, with the requirement of legs to induce the movement of the rotor [8], as well as the proper mixing of two standing waves at the same frequency, with either a bending and a longitudinal mode [9] or two bending modes [10].

In the field of mobile robots based on piezoelectric motors, the literature is more recent. Different reports combined piezoelectric materials with legs to attain locomotion [11,12,13]. For those robots whose movement relies on the generation of stationary waves, millimeter-sized legged devices have already been reported [14,15,16]. Furthermore, in the case of traveling waves, the state of the art involves the locomotion of 180-mm long plates actuated by piezoelectric patches, without using legs [17,18]. Taking these as the background, the present work aimed to further develop the traveling-wave-based locomotion of miniature plates with two piezoelectric patches. Miniaturization was accomplished by reducing the size of the structure down to 20 mm long and 3 mm wide. Furthermore, to pursue future untethered applications, the maximum applied voltage was limited to 65 V peak-to-peak (V_pp_). This voltage limitation resulted in no locomotion of the bare structure, requiring the use of legs to induce movement at a voltage as low as 20 V_pp_. In addition, the size and location of the patches that maximize the vertical displacement of the plate, while preserving the progressing nature of the generated wave, were calculated with the help of a 1D analytical model of the patches/plate system. This study completes the work begun in a previous study based on numerical finite element analysis [19], where the transversal displacement along the structure was analyzed for five different locations of a fixed-length patch. All these improvements are expected to reduce the miniaturization limits in the field of mobile robots.

## 2. Device Design

The first part of this section considers the effect of the device design and the excitation signals on the generation of a traveling wave in a plate with two piezoelectric patches. The electromechanical performance of piezoelectric layers on laminates was already well established by Lee and Moon’s seminal work [20]. Later reports demonstrated the generation of linear traveling waves on a beam using external actuation forces [21,22], or with two piezoelectric patches on the same beam [23], by combining two vibration modes with appropriate amplitudes and phases. In the latter, the patches were symmetrically situated with respect to the center of the plate, where each of them was actuated with the same sinusoidal signal, but the phase was shifted. Here, we used a similar approach to design the robotic structures to be discussed in the next sections.

**Figure 1:**
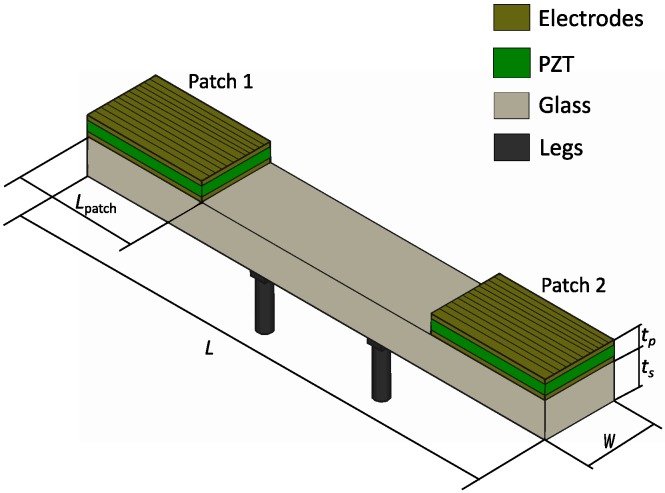
Representation of the fabricated structure. *t_p_* and *t_s_* are the thicknesses of the piezoelectric film and the substrate, respectively, *W* is the width of the structure, *L* is the length of the structure, and *L*_patch_ is the length of the patch.

Figure 1 shows the schematic of the structure under study. It consisted of a supporting layer of glass with a length of 20 mm, width of 3 mm, and thickness of 1 mm. Piezoelectric patches of electroded lead zirconate titanate (PZT), 0.2 mm thick, covered a length *L*_patch_. The thickness of the electrodes was neglected in the model. The patches started at the edges of the glass and both covered a given length to be determined by design, creating a symmetric configuration with respect to the center of the structure.

According to the basic mode superposition [24], the vertical displacement at any time and position along the length of the structure can be expressed as:

(1) $$v\left(x,t\right)=\overset{\infty }{\underset{i=1}{\sum }}\varphi_{i}\left(x\right)\times T_{i}\left(t\right)$$

where $\varphi_{i}$ are the shapes of the normalized flexural modes and $T_{i}$ are the time-dependent modal coefficients. The previous expression can be truncated to the modes nearest to, above, and below the actuation frequency. The time-dependent modal factor can be Fourier-transformed to the following expression for each of the patches with an actuation voltage $Ve^{j\left(\omega t+\phi \right)}$ in the complex domain [20,23]:

(2) $$T_{i}\left(\omega \right)=-\frac{Y_{p}d_{31}\left(\frac{w}{2}\right)t_{p}\left(t_{p}+t_{s}-2z_{n}\right)\left({\varphi^{\prime }}_{i}\left(l1\right)-{\varphi^{\prime }}_{i}\left(l2\right)\right)}{t_{p}\left(\omega_{i}^{2}-\omega^{2}+2j\zeta \omega \omega_{i}\right)}Ve^{j\phi },$$

which is given by the product of the modal admittance and the complex amplitude of the actuation voltage, where $Y_{p}$ is the Young modulus of the piezoelectric film; *d*_31_ is the piezoelectric coefficient; $z_{n}$ is the neutral axis of the laminate structure; $\varphi^{\prime }$ is the first spatial derivative of the modal shape; l1 and l2 are the initial and final position of the patch, respectively; ω is the frequency of actuation; $\omega_{i}$ is the resonance frequency of mode *i*; and ζ is the damping ratio. The traveling wave (TW) envelope is taken as the magnitude of *v*(*x*,*ω*) for each position *x* at a given frequency. To simplify the calculation, the modal shapes and resonance frequencies were obtained analytically assuming a constant cross-section along the length of the structure, with the PZT covering the entire glass and the electrodes limited to the extension of the patch [25].

Regarding the parameters of the model, the amplitude of the voltage applied to the patches was 10 V, the *d*_31_ piezoelectric coefficient of PZT was 180 pm/V, and the damping factor of the modes was equal to 0.001, corresponding to a quality factor of 500. Table 1 shows the rest of the parameters.

We then considered two figures of merit: (i) standing wave ratio (SWR), defined as the ratio of the maximum to the minimum value of the TW envelope, which is related to the quality of the traveling wave: the closer to 1, the better the traveling wave; and (ii) the average displacement of the TW envelope, named <TW>, which is associated with the speed or energy of the wave. In the rest of the section, SWR and <TW> are taken in a central window of the total length corresponding to 60% to remove the effect of the boundary conditions at the edges.

This approach resulted in three key variables that may be varied to improve the quality and amplitude of the TW: patch length, frequency of actuation, and phase shift between the sinusoidal signals on each of the patches. Malladi et al. [23] reported the effect of the frequency of actuation and the phase shift. Here, we also considered the effect of the patch length on the two figures of merit mentioned before.

**Figure 2:**
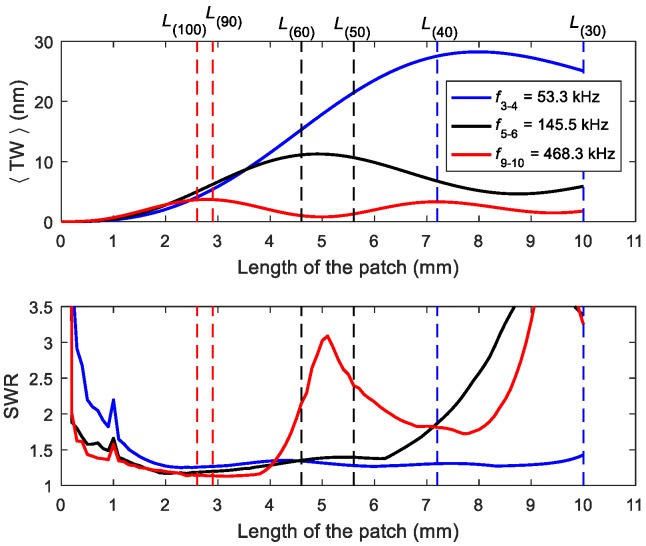
Average displacement of the traveling wave envelope (<TW>) and standing wave ratio (SWR) as a function of the length of the patch for three different frequencies of actuation *f*_3–4_, *f*_5–6_, and *f*_9–10_, and a phase shift of 90° between patches. *f*_m–n_: mid-frequency between modes (m0) and (n0). The vertical line *L*_(n0)_ represents the first zero, excluding the edge of the plate, of the second derivative of the modal shape (n0), where n will vary depending on the mode under consideration.

First, we studied the dependence of SWR and <TW> on the patch length with a fixed frequency of actuation (average of the two consecutive resonant frequencies) and phase shift (90°). Figure 2 shows the results. Leissa´s nomenclature is used to identify the modes of vibration (the first digit is the number of nodal lines along the length of the plate and the second digit is the number of nodal lines along the width of the plate) [25]. Three different frequencies of actuation were considered depending on the couple of modes to be mixed: half-way between modes (30) and (40), (50) and (60), and (90) and (100); named *f*_3–4_, *f*_5–6_, and *f*_9–10_, respectively. The effect of varying these two variables, namely frequency and phase, is shown below.

There was a clear dependence of the figures of merit on the length of the patch, and for each of the frequencies, there was an optimal length of patch *L*_m–n_, where m refers to mode (m0) and n to mode (n0), that provided a maximum of <TW>: *L*_3–4_ = 8.3 mm for *f*_3–4_, *L*_5–6_ = 4.9 mm for *f*_5–6_, *L*_9–10_ = 2.8 mm for *f*_9–10_, while maintaining a value for the SWR close to the ideal value of 1.

Furthermore, the information of Figure 2 was correlated with the second derivative of the modal shape along the length of the structure. As already reported, optimal actuation of a given mode can be attained using an electrode distribution that covers only the regions of the surface where the sign of the second derivative of the mode shape (associated with stress on the surface) is either positive or negative [26]. The dashed vertical lines in Figure 2 represent the position of the first zero (excluding the edge of the structure) of the second derivative of the three pairs of modal shapes involved in the calculations. A patch of length *L*_(n0)_, where n will vary depending on the mode under consideration, would optimally actuate this individual mode (n0). Note that the optimum patch length for the TW generation with modes m–n, *L*_m–n,_ lays half-way between lengths *L*_(m0)_ and *L*_(n0)_ as a balance between the optimal patches for each individual mode contributing the most to the TW: *L*_m–n_ ≈ (*L*_(m0)_ + *L*_(n0)_)/2. Therefore, as a rule of thumb, the second derivative of the modal shapes can be used to determine the best patch length without requiring any complex simulation approach.

**Figure 3:**
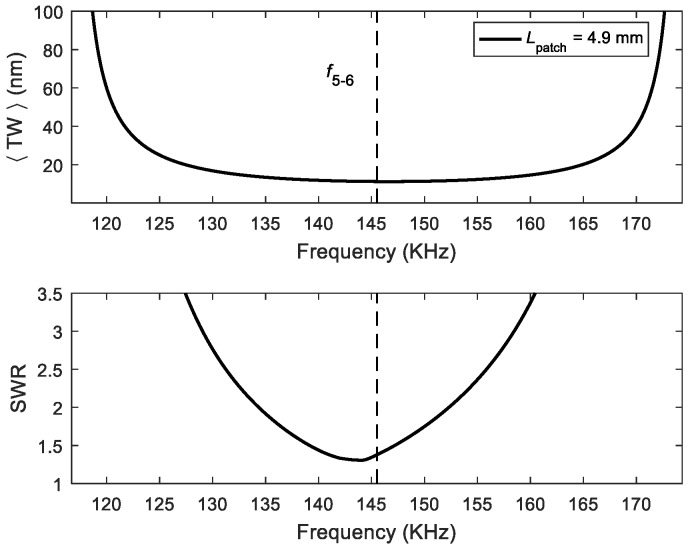
<TW> and SWR as a function of the frequency of actuation for the optimum patch length *L*_5–6_ and a phase shift of 90° between the signals applied to the patches.

For the optimum patch length deduced before, we then considered whether the figures of merit could be improved by varying the frequency of actuation while maintaining the 90° phase shift. Figure 3 shows the results for the combination of modes (50) and (60) as an example. The best SWR was very close to the mid-frequency *f*_5–6_, and the displacement varied only slightly; therefore, there was very little room for improvement by changing the frequency around the mid-frequency under these conditions. This conclusion was also reached for other combinations of modes.

**Figure 4:**
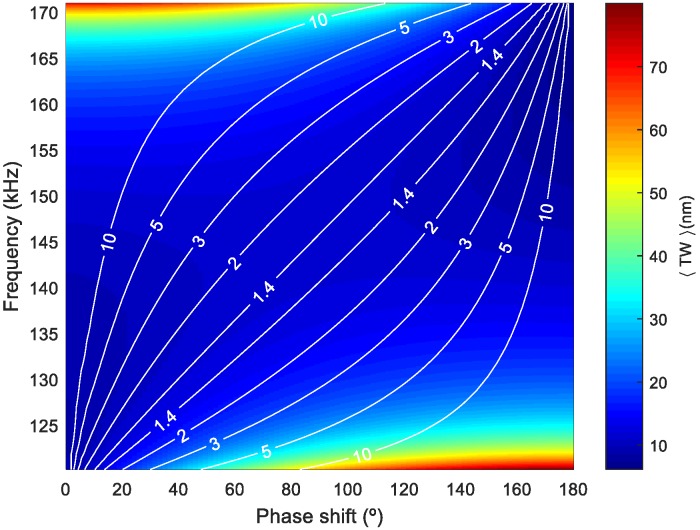
Surface plot representation of <TW> for the combination of modes (50) and (60) as a function of actuation frequency and phase shift, with white isolines corresponding to different values of SWR. The <TW> value is represented by the color scale.

**Figure 5:**
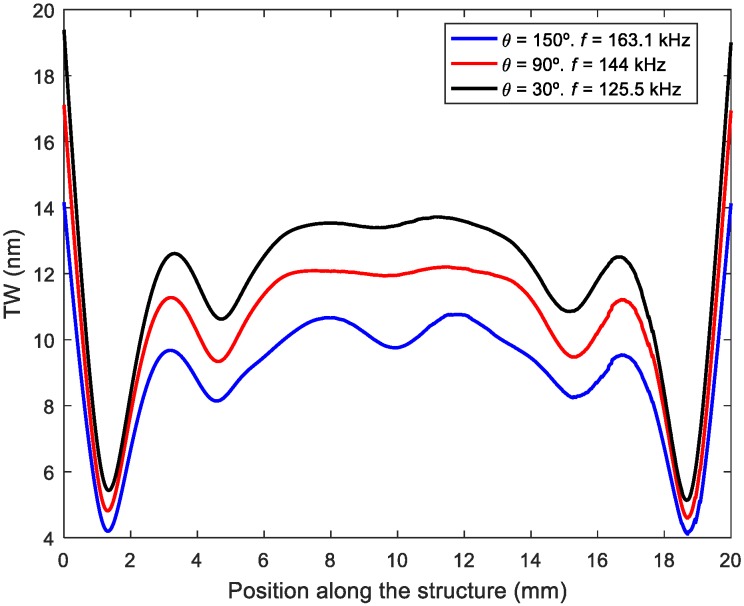
Envelope of the TW along the length of the structure for the combination of modes (50) and (60) under three different conditions of frequency of actuation *f* and phase shift *θ*. The optimal patch length *L*_5–6_ was used.

Now we focus on the effect of both the frequency of actuation and the phase shift for the optimal patch length. Figure 4 shows a surface plot of <TW>, corresponding to the combination of modes (50) and (60), as a function of frequency and phase shift as a color map for different SWR isolines. It can be seen that an increase in <TW> was only possible by deteriorating the SWR and that there were many possible couples of frequency and phase leading to a similar <TW> and SWR, as shown with the isoline corresponding to SWR = 1.4, for example. This is crucial for avoiding undesirable torsional modes that might be in between the two flexural modes under study and hinder the TW generation based on the two-mode-approximation. This was further illustrated by plotting the envelope of the TW for the mixing of modes (50) and (60) under three different combinations of frequency and phase shift (Figure 5).

Once the design guidelines for the patch size, the actuation frequency, and the phase shift were decided upon, legs were included in the design. This addition was experimentally required to observe stable locomotion of the structures at voltages below the maximum applied, which was 65 V_pp_. This differed from previous studies [17,18], where locomotion was attained without the help of legs. We attributed this need for legs to the smaller size and weight in our case, as well as to the limit imposed on the maximum voltage applied. Legs amplify the horizontal displacement [27], and at the same time, confine the point of contact to specific areas of the robot. A pertinent question here is where to locate the legs on the glass plate. A key design goal of propagating-wave-based locomotion is the elliptical trajectory of the surface particles of the elastic body due to the coupling of longitudinal and transverse motions with the appropriate phases to achieve bidirectionality using the 90° shift of the actuation signals. Therefore, the leg positions should coincide with those surface points where elliptical trajectories are realized. To determine those locations on the structure, we note that the vertical displacement v(x,t) along the plate can be expressed as follows:

(3) v(x,t)=A(x)·cos(ωt+θ(x)),

which resembles a pure TW, but instead of a constant amplitude, we have a position-dependent amplitude A(x) (the envelope of the TW defined above), and instead of a phase term proportional to the position, there is a general function of the phase θ(x). Furthermore, the horizontal displacement, *u*, at the bottom face of the plate where the legs are to be placed is [28]:

(4) $$u\left(x,t\right)=-h\frac{\partial v\left(x,t\right)}{\partial x}=-h\left[\frac{dA\left(x\right)}{dx}\cdot \cos\left(\omega t+\theta \left(x\right)\right)-A\left(x\right)\cdot \frac{d\theta \left(x\right)}{dx}\cdot \sin\left(\omega t+\theta \left(x\right)\right)\right],$$

where h is the thickness between the neutral plane of the structure and the bottom face of the plate. For the displacement at the tip of the leg, the same expression holds, but the length of the leg is added to the thickness h.

According to the two previous expressions, only those positions along the length of the structure where the derivative of A(x) is null (local maximum or minimum) will present an elliptical trajectory. That is to say, at those positions where the TW envelope is almost constant, the surface particles, and hence the tip of the legs, will describe elliptical trajectories. By returning to Figure 5, we notice that the positions to consider were those located at the central plateau of the TW envelope. If the legs are located where the derivative of A(x) is significant, a linear-like displacement is then obtained, as for standing wave linear motors.

## 3. Materials and Methods

**Figure 6:**
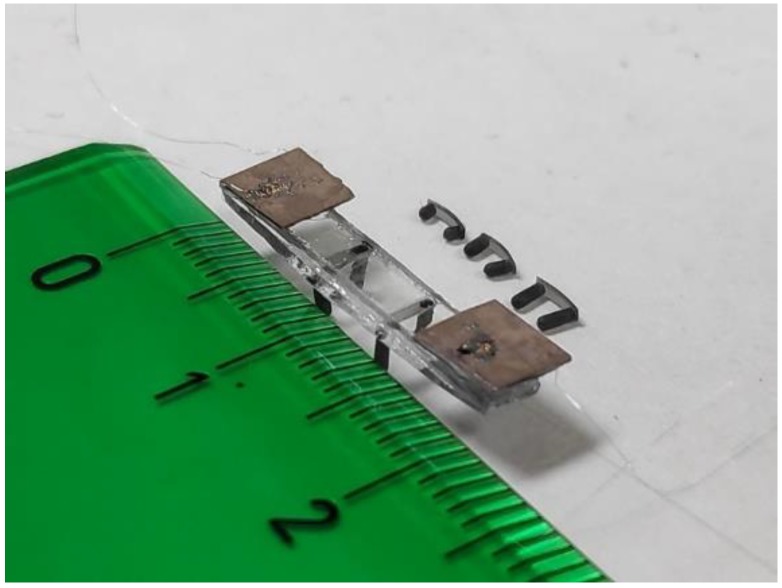
Photograph of a legged structure and U-shaped pairs of legs of 0.5, 1, and 1.5 mm. The numbered ruler marks represent centimeters.

Next, we focus our attention on the fabrication procedure. Figure 6 shows a picture of one of the fabricated structures. A piece of glass with a length of 20 mm, width of 3 mm, and thickness of 1 mm was obtained from a glass slide (VWR International, Radnor, PA, USA) via machine (Buehler, Lake Bluff, IL, USA) drilling. Two PZT patches (PI Ceramic GmbH, Lederhose, Germany) with a thickness of 200 μm and a width of 3.5 mm (slightly larger than the plate to allow for contact to the bottom face) were glued to the glass using a cyanoacrylate adhesive (Loctite, Düsseldorf, Germany). The actual length of the fabricated patches was 5 mm, close to the optimum value of 4.9 determined in Figure 2, for an efficient actuation of modes (50) and (60). The robots were powered externally using 25-micron wires connected to the piezoelectric patches.

U-shaped pairs of legs were 3D printed with a stereolithography (SLA) B9 Core printer (B9Creations, Rapid City, SD, USA) using a material named Black Resin (B9Creations, Rapid City, SD, USA) (see Figure 6). The pair of legs were glued along the width of the plate. Samples with two and three pairs of legs were fabricated. In the case of the two pairs of legs, these were located at the edges of the TW envelope plateau mentioned earlier. For the three pairs of legs, the third pair was located at the center of the structure. The shape of the legs was cylindrical, 0.6 mm in diameter, with varying lengths from 0.5 mm to 1.5 mm. The mass of the robot was about 240 mg.

## 4. Results

**Figure 7:**
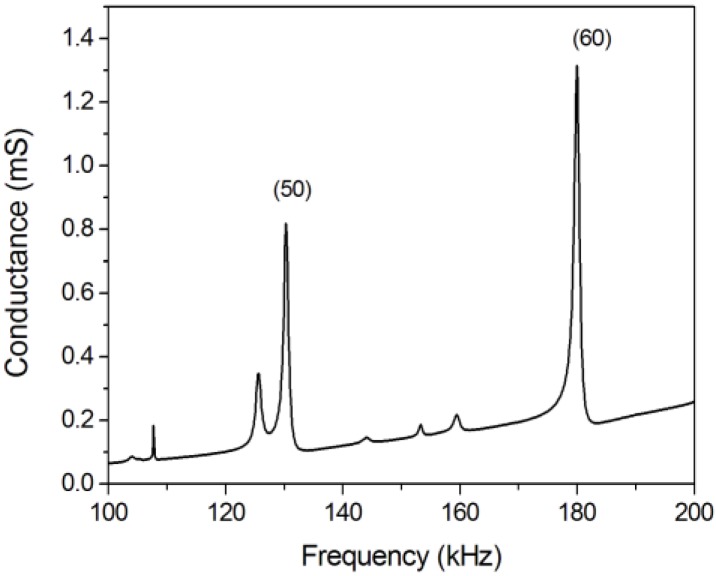
Electrical conductance of a robot with 0.5-mm legs.

Figure 7 shows the electrical conductance of a robot with 0.5-mm legs. Two peaks can be clearly identified corresponding to modes (50) and (60). Figure S1, included in the Supplementary Materials, compares the conductance of this device with and without legs. There was almost no difference between the two measurements, which corroborates the negligible impact of the legs on the standing waves corresponding to the modes. Once the resonant frequencies were known, the frequency of actuation was adjusted manually. For this sample, the actuation frequency was set to 161 kHz, close to the mid-frequency between the measured modes (50) and (60). It is important to notice that the frequency of actuation, 161 kHz, differed from the estimated frequency *f*_5–6_, by just 10%. This difference might be attributed to the limitations of the 1D model at representing the 3D structure of the robot, as well as to uncertainties in the mechanical parameters of the materials. Table S1 of the Supplementary Materials compares the resonant frequency of different modes, found using both experimentation and calculated using the 1D model and a 3D finite element analysis. It also shows the values for *L*_(n0)_, which is the first zero of the second derivative of the modal shapes deduced by the 1D and 3D models.

**Figure 8:**
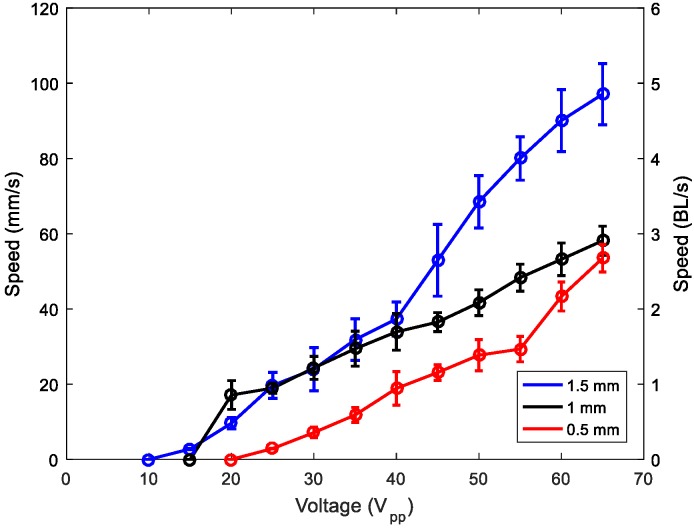
Speed of the robots versus applied voltage for legs with different lengths: 0.5 mm (red), 1 mm (black), and 1.5 mm (blue). Circles represent experimental data and are joined with lines for guidance purposes. BL/s: Body lengths per second.

Next, we present the characterization of the fabricated robots in terms of speed and force. Figure 8 shows the speed of the robot versus the applied voltage. Ten measurements were taken at each voltage and the standard deviation was about ±3.5 mm/s. The phase shift between patches was fixed to either 90° or −90° to confirm the bidirectional movement. Video S1, included with the Supplementary Materials, shows how the direction was reversed by changing the phase. The set-up for the speed measurement consisted of two infrared LEDs separated by 100 mm, where each was aligned with a photodiode. The set-up allowed for measurement of the time required by the robot to travel 100 mm along a rail on glass by tracking the light interruption events when the robot passed below the infrared LEDs with a frequency counter. Robots with legs of 1.5 mm showed a less uniform speed, with difficulties in maintaining the rectilinear displacement, which might be related to the interference of an intrinsic mode of vibration of the legs. The modes of vibration of 0.5- and 1-mm legs were far away from the frequency of actuation. When comparing robots with 0.5- and 1-mm legs, a better performance was observed for the 1-mm leg, which might be attributed to an enhancement of the horizontal displacement at the tip of the leg, as mentioned previously. For the maximum voltage applied, namely 65 V_pp_, the velocity for the 1-mm-legged structure reached 60 mm/s, which was equivalent to 3 BL/s (body lengths per second). These results are comparable to the state-of-the-art in miniature soft robotics, with performances similar to arthropods [29]. Furthermore, notice that the minimum voltage required to initiate movement with 1-mm legs was as low as 20 V_pp_, which might facilitate the implementation of an untethered robot with an integrated driving signal.

**Figure 9:**
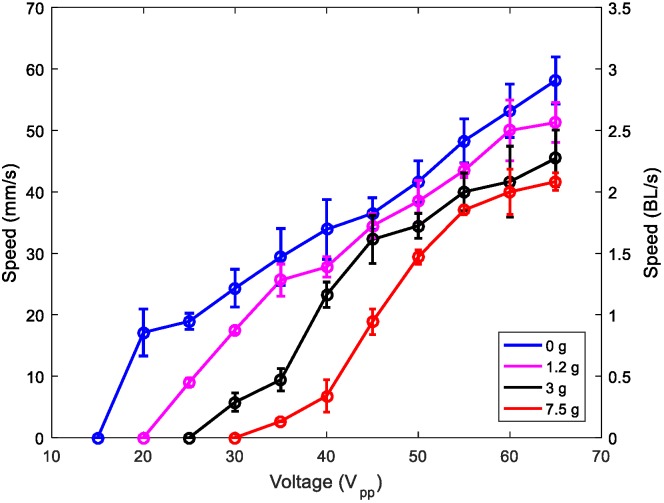
Speed of the 1-mm-legged robot versus applied voltage for different masses: no load (blue), 1.2 g (pink), 3 g (black), and 7.5 g (red).

Furthermore, we investigated the effect of mass loading on the performance of the robot. Figure 9 shows the speed versus applied voltage for different loading masses. The robot carried a mass of 7.5 g, which was 40 times its weight, at a speed of about 40 mm/s at the maximum voltage applied. This result shows the potential to incorporate electronic circuits on board, for communication, control, sensing, or other applications. Video S2 shows the locomotion with a mass of 7.5 g.

**Figure 10:**
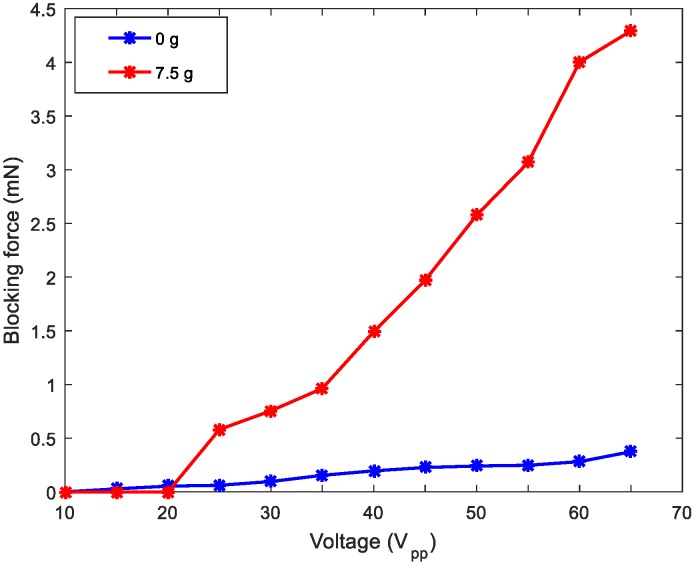
Blocking force of the robot for different masses: no load mass (blue) and 7.5 g (red).

To complete the characterization of the robot, Figure 10 displays the blocking force under different mass loadings. The force was measured while the robot contacted a force sensor (Honeywell FSG Series, Morris Plains, NJ, USA) with the actuation voltage applied. As expected, the blocking force increased as the mass loading increased [30].

**Figure 11:**
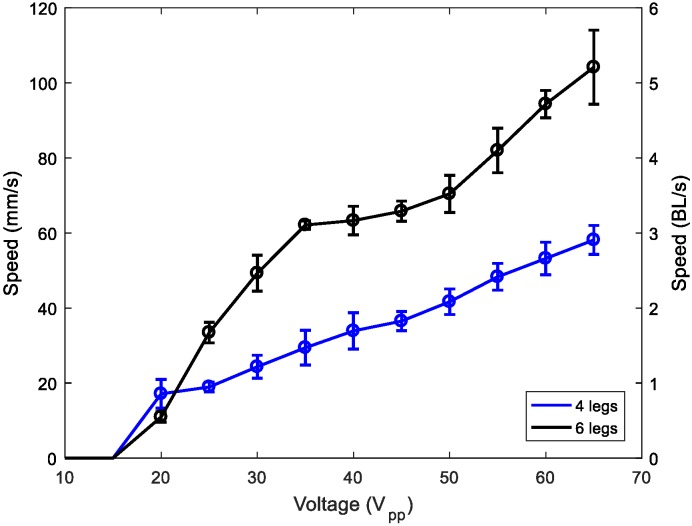
Speed of the 1-mm-legged robot versus applied voltage for two (blue) and three (black) pairs of legs.

Finally, Figure 11 shows the comparison between two and three pairs of 1-mm legs. A clear improvement can be seen when using three pairs of legs, with a speed as high as 5 BL/s. Further investigations are in progress to study the effect of increasing the number of legs.

## 5. Conclusions

This paper contributes to the development of miniature mobile robots based on TWs generated by the actuation of symmetrically located piezoelectric patches. Guidelines were proposed for the design of the patches and the driving signals. 3D printed legs were implemented in our devices, which is an approach commonly restricted to standing-wave-based systems. The combination of the optimal patch length and legs resulted in a mobile rigid robot with a speed of 5 BL/s at a voltage of 65 V_pp_, with the capability of transporting 40 times its weight.

## Figures and Tables

**Table 1:** Structural properties of the materials.

Material	Thickness (mm)	Young’s Modulus (GPa)	Density (kg/m^3^)
Glass	1	72.9	3350
PZT	0.2	62	7800

## Supplementary Materials

The following are available online at https://www.mdpi.com/2072-666X/11/3/321/s1, Figure S1: Electrical conductance of a 0.5-mm-legged robot compared with the case without legs. Table S1: Resonant frequency and position of the first zero of the second derivative of the shape of modes (40), (50), (60), and (70) for three cases: experiment, 1D analytical model, and 3D finite element analysis. Video S1: Bidirectional locomotion of a 1-mm-legged structure with no load. Video S2: Bidirectional locomotion of a 1-mm-legged structure with a load of 7.5 g.
