# Supplementary material for: Motion of a Legged Bidirectional Miniature Piezoelectric Robot Based on Traveling Wave Generation
Source: Micromachines (Basel). 2020 Mar 20;11(3):321. doi: 10.3390/mi11030321 (PMC7142472; doi:10.3390/mi11030321)
Supplement: Supplementary file 1 [file micromachines-11-00321-s001.zip › micromachines-739834-supplementary materias-for final.pdf]

micromachines

*Supplementary Materials*

# Motion of a Legged Bidirectional Miniature Piezoelectric Robot Based on Traveling Wave Generation

**Jorge Hernando-García, Jose Luis García-Caraballo, Víctor Ruiz-Díez and Jose Luis Sánchez-Rojas**

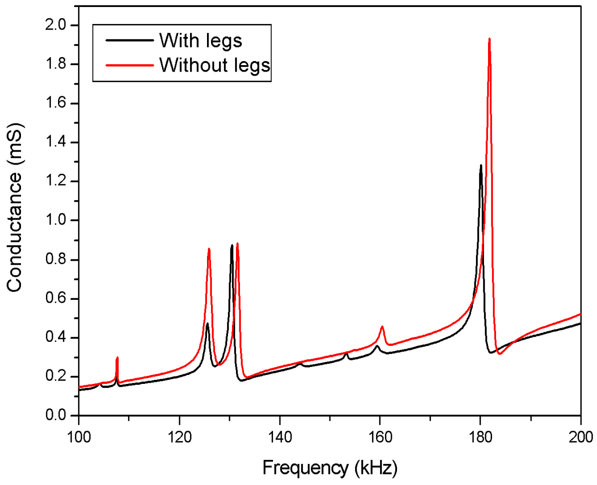


**Figure S1.** Electrical conductance of a 0.5 mm-legs robot compared with the case without legs.

Table S1. Upper part: Resonant frequency of modes (40), (50), (60) and (70) for three cases: experiment, 1D analytical model and 3D finite element analysis (FEM). Lower part: Position of the first zero of the second derivative of the modal shapes, $L_{(n0)}$, where n depends on the mode under consideration, as deduced by the 1D and 3D models.

For the resonant frequencies, there is a discrepancy between the 1D and the 3D models, as might be expected. Besides, the difference between the experiment and the 3D FEM model could be corrected by uncertainties in the values of the mechanical properties of the materials. It is important to emphasize that the value of $L_{(n0)}$, corresponding to the position of the first zero of the second derivative of the modal shape, is very similar for the simple 1D model and the precise 3D model, what supports the use of the 1D model for a clear identification of the optimal patch.

**Table S1.** Resonant frequency and position of the first zero of the second derivative of the shape of modes (40), (50), (60) and (70) for three cases: experiment, 1D analytical model and 3D finite element analysis.

| **Mode** | $f_{(40)}$ **(kHz)** | $f_{(50)}$ **(kHz)** | $f_{(60)}$ **(kHz)** | $f_{(70)}$ **(kHz)** |
|---|---|---|---|---|
| Experiment | 85,4 | 130,2 | 180 | 248,4 |
| 1D analytical | 78,1 | 116,7 | 174,3 | 243,5 |
| 3D FEM | 61,8 | 102,1 | 146,5 | 193 |

| Mode | $L_{(40)}$ (mm) | $L_{(50)}$ (mm) | $L_{(60)}$ (mm) | $L_{(70)}$ (mm) |
|---|---|---|---|---|
| 3D FEM | 7,3 | 5,7 | 4,7 | 3,9 |
| 1D analytical | 7,2 | 5,6 | 4,6 | 3,8 |
